# Immunomodulatory potential of probiotics oraly delivered with β- LG to Balb/C mice

**DOI:** 10.1186/2045-7022-5-S3-P121

**Published:** 2015-03-30

**Authors:** Dagmara Złotkowska, Ewa Wasilewska, Justyna Chudzik-Kozłowska

**Affiliations:** 1 Department of Food Immunology and Microbiology, Division of Food Science, Institute of Animal Reproduction and Food Research of PAS, Olsztyn, Poland

## 

**Figure 1:**
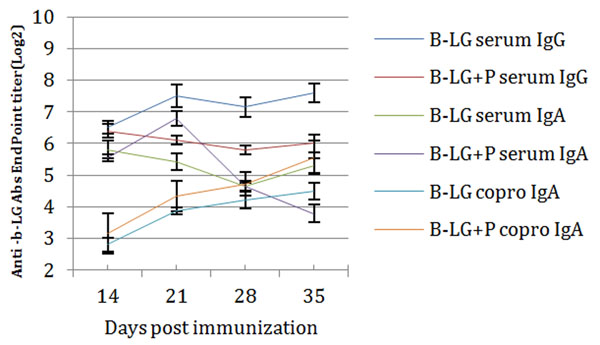
Specific antibodies titer

Two commercial probiotics preparations were analysed regarding the possibility to induce different T cells population. Determining CD4+, CD4+CD8+ and CD8+ expression (Tab.1) one was subjected to further experiments and delivered it together with milk allergen β-LG to Balb/C mice. 14, 21, 28 and 35d from the beginning of experiments specific serum IgG&IgA, sIgA and copro-IgA were checked. We found significant decrease of humoral response in group fed with β-LG with probiotic compared to group with β-LG only, the same time increase specific copro-IgA by 35 days of experiments was observed (Fig. 1). Flow cytometry analysis of T cells subsets show significant decreased in percentage of CD8+ population in head and neck lymph nodes (HNLN) and spleen (SPL) when probiotic was delivered. In Peyer's Patches elevated number of CD4+CD8+ and CD8+ T cell subsets was observed in the same group. During *in vitro* studies lymphocytes isolated from group β-LG+probiotic show significant increased CD8+, CD8+CD4+ compare to B-LG group. Results show that probiotics has potential to modulate immune answer to food allergens.

**Table 1:** 

		DICOFLOR	SD	PROACTIVE	SD
SPL	CD8	9.96	0.766	9.2	1.3

	CD4CD8	0.276	0.146	1.19	0.567

	CD4	23.9	1.62	21.7	0.808

PP	CD8	2.81	1.41	1.02	0.639

	CD4CD8	0.26	0.149	0.609	0.914

	CD4	12.2	4.7	3.19	0.75

HNLN	CD8	17.6	0.952	18.1	2.12

	CD4CD8	1.26	0.534	0.323	0.203

	CD4	47.5	3.01	43.4	2.83

MLN	CD8	13.2	1.59	14.8	1.53

	CD4CD8	1.1	1.14	0.842	1.04

	CD4	45.4	14.6	52.3	4.08

SERUM	CD8	11.7	1.01	9.18	0.479

	CD4CD8	0.281	0.463	0.0554	0.0213

	CD4	35.6	3.42	29	2.87

